# Minimal Gene Regulatory Circuits for a Lysis-Lysogeny Choice in the Presence of Noise

**DOI:** 10.1371/journal.pone.0015037

**Published:** 2010-12-20

**Authors:** Mikkel Avlund, Sandeep Krishna, Szabolcs Semsey, Ian B. Dodd, Kim Sneppen

**Affiliations:** 1 Center for Models of Life, Niels Bohr Institute, Copenhagen, Denmark; 2 National Centre for Biological Sciences, Bangalore, India; 3 Molecular and Biomedical Sciences (Biochemistry), University of Adelaide, Adelaide, Australia; Baylor College of Medicine, United States of America

## Abstract

Gene regulatory networks (GRNs) that make reliable decisions should have design features to cope with random fluctuations in the levels or activities of biological molecules. The phage 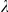 GRN makes a lysis-lysogeny decision informed by the number of phages infecting the cell. To analyse the design of decision making GRNs, we generated random *in silico* GRNs comprised of two or three transcriptional regulators and selected those able to perform a 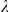-like decision in the presence of noise. Various two-protein networks analogous to the 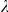 CI-Cro GRN worked in noise-less conditions but failed when noise was introduced. Adding a 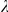 CII-like protein significantly improved robustness to noise. CII relieves the CI-like protein of its ‘decider’ function, allowing CI to be optimized as a decision ‘maintainer’. CII's lysogenic decider function was improved by its instability and rapid removal once the decision was taken, preventing its interference with maintenance. A more reliable decision also resulted from simulated co-transcription of the genes for CII and the Cro-like protein, which correlates fluctuations in these opposing decider functions and makes their ratio less noisy. Thus, the 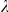 decision network contains design features for reducing and resisting noise.

## Introduction

Biological molecules are subject to random fluctuations in the rates of their synthesis, distribution, activity and decay [1]–[4]. This noise becomes more significant as the number of molecules involved becomes small and can potentially interfere with the efficient functioning of gene regulatory networks (GRNs). GRNs that must make reliable developmental decisions are presumably designed to minimize noise but it is not well understood how this is achieved. Here we examine how noise is reduced in *in silico* GRNs selected to reliably perform an informed lysis-lysogeny decision like that made by bacteriophage 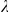 [5], [6].

A standard approach in *in silico* biology is to fit parameters to molecular mechanisms in order to reproduce observed features. While this approach often gives useful insights into particular regulatory systems, it only provides a limited understanding of why a given regulatory network has a specific structure. Many models of this type have been used to analyse the developmental decision of the 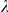 bistable GRN [7]–[11]. Another, and less explored approach is to assume a toolbox of available gene regulatory mechanisms, and then sample many combinations of these to test how well they perform a given task [12]–[14]. In this case the success criterion is not only whether a certain network structure can function properly with a given set of parameters that describe the strengths of the regulatory links but also how easy it is to find such parameters. A successful network structure is one which is robust to variations in these parameters [15]. Previously, [16] we used this second approach to explore small network structures, or motifs, that are able to mimic the ability of the 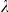 network to count, that is, to make a decision between lysis and lysogeny based on the number of phages infecting the cell (multiplicity of infection, MOI) [17]–[21], a task complicated by replication of the phage genome soon after infection [22].

Avlund et al. [16] constructed *in silico* 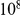 different two-node transcriptional networks and used deterministic simulations to test the ability of these networks to choose a stable ‘lytic’ state after a single infection (MOI = 1) or a stable ‘lysogenic’ state after a double infection (MOI = 2). Given the right parameters, any motif containing mutual repression by the two proteins could be made to count correctly and remember the decision. This core motif is analogous to the CI-Cro mutual repression in 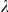 and other bacteriophages that creates a bistable positive feedback loop [23]–[26]. This finding was surprising because 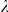
's ability to count is believed to require other proteins, particularly CII, a lytically expressed protein which fosters lysogeny by activating transcription of CI [6].

Here we extend this study by testing the ability of the networks to function in the presence of gene expression noise. We find that the two-node transcriptional networks are fragile; very few parameter sets allow these motifs to function in a noise-resistant way. Thus, it seems unlikely that 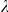 or other phages can use a CI-Cro system to count reliably. However, when a CII-like function is added to the motifs it is much easier to find noise-resistant networks, supporting the idea that CII has an important role in counting by 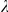. Analysis of the successful networks identifies strategies that are employed by lambda to make a reliable developmental decision.

## Results and Discussion

### Approach

**Figure 1:**
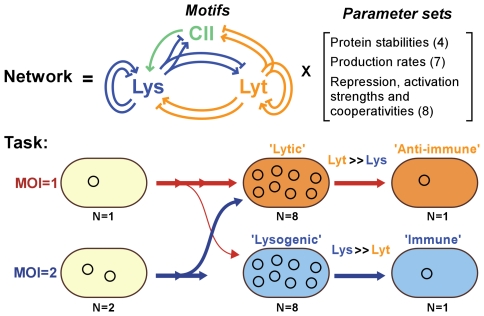
Construction and testing of in silico networks for performance in a 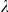-like counting and memory task. Each network was a combination of a network motif and a random parameter set. Each motif was one of the 80 combinations of transcriptional inter- and auto-regulation by two proteins, Lys (lysogenic) and Lyt (lytic), with each regulatory link either activatory, repressive or absent. In the two-protein networks only Lys and Lyt are present; in the two-protein+CII networks, a 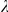 CII-like function was added. A set of 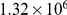 random parameter sets were generated and used in each network motif. In the deterministic counting and memory task, ‘infection’ by a single copy of the network followed by replication should always produce the ‘lytic’ (Lyt 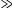 Lys) outcome, while infection by two copies should always produce the reverse outcome. In the stochastic counting task, where noise is added, some transition from one pathway to the other is permitted. In both cases ‘memory’ is required; the lytic or lysogenic states should remain stable when the copy number is reduced to one.

The two-protein networks constructed by Avlund et al. [16] consist of the Lys (for lysogenic) and Lyt (for lytic) proteins, each potentially able to regulate their own gene or the gene for the other protein (the genes *lys* and *lyt*). Lys is analogous to 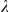 CI and Lyt to Cro. Each regulatory link was either repression, activation or an absence of regulation, giving 80 possible 2-protein architectures or motifs (Figure 1). Avlund et al. also made 2-protein+CII networks in which a 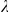 CII-like function was added to each of these motifs as a protein that adds to Lys production by activating transcription from a separate promoter. Like the CII stimulated 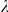 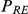 promoter, this promoter was not regulated by Lys or Lyt and had almost no basal activity [27]. The *cII* and *lyt* genes were regulated identically (Figure 1) to mimic co-transcription of *cII* and *cro* in 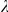.

Avlund et al. created 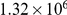 sets of parameters for the 2-protein+CII network (i.e. providing for all links). Each set included randomly chosen parameters setting the basal and maximal activity of each gene, the strength and cooperativity of repressive and activatory links, and first-order protein degradation rates (Figure 1 and Methods). In addition, a parameter was included to provide for decreased degradation of CII at higher CII concentrations, as seen for 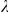 [27], [28]. Each parameter set was applied to each of the 80 two-protein motifs (ignoring the parameters for links that were not present in the particular motif), generating over 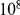 two-protein networks. The same parameter sets were also applied to each of the two-protein+CII motifs.

Each network was tested in simulated MOI = 1 and MOI = 2 infections (one or two initial copies of the network ‘genome’) in which the initial protein concentrations were set to zero. In Avlund et al. [16], the change in protein concentrations over time occurred deterministically. Here, we introduced stochasticity in the rates of protein production and degradation using the Gillespie algorithm (see Methods). Co-transcription of the *cro* and *cII* genes in 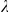 was simulated by making the production bursts of Lyt and CII synchronous.

We carried out 100 simulated infections for the MOI = 1 and MOI = 2 conditions to obtain a reasonable sample of possible outcomes. As in Avlund et al. [16], replication of the genome was simulated by a doubling of the number of network copies with a fixed generation time, 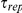, with the genome allowed to replicate to 8 copies, as we assume that the decision between lysis and lysogeny is made by this point. A successful network had to be able to take two clearly different regulatory trajectories, equivalent to lytic and lysogenic development. The existence of two distinct states was defined by the ratios of the concentrations of the Lys and Lyt proteins, with a high Lys/Lyt ratio indicating lysogeny and a low Lys/Lyt ratio indicating lysis. To test for such stable and distinct states we continued the simulation for 20 generation times, without further replication, and examined the Lys/Lyt ratios (see Methods). We also required that the regulatory states remained distinct when the genome was reduced to single copy – ‘memory’ of the decision (Figure 1). This reflects the ability of a single copy prophage in a lysogenic cell to be able to maintain the immune state, as well as the ability of the system to exist in a stable, single-copy ‘lytic’ or anti-immune state, as seen for many phages (e.g. [23]–[25]).

In the deterministic simulations the network was scored as successful if it produced the lytic outcome in MOI = 1 infections and the lysogenic outcome in MOI = 2 infections. In the stochastic simulations success required the lytic outcome in at least 98% of MOI = 1 infections and the lysogenic outcome in at least 60% of MOI = 2 infections. These frequencies are similar to those observed by Kourilsky (see the analysis in [29]) in which more than 99% of single infections go lytic, while 69% of the double infections go lysogenic. We note that higher frequencies of lysogeny after single infections have been observed in more recent experiments [19], [21].

### Two-protein networks are fragile to noise

**Figure 2:**
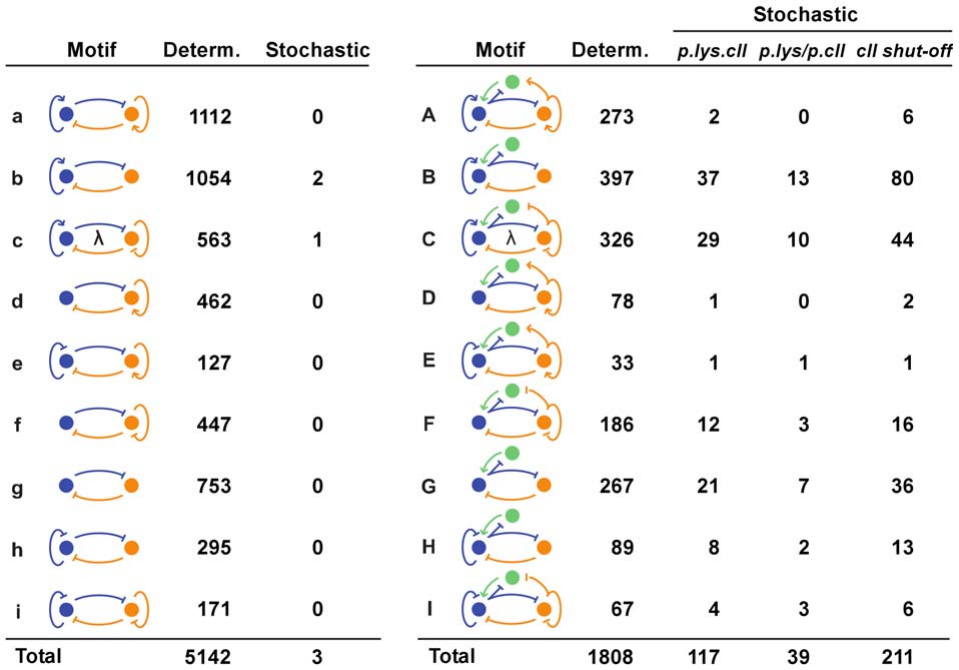
Two-protein and two-protein+CII networks that can perform the deterministic counting and memory task and their performance under stochastic simulations. In the 2-protein stochastic simulations and the two-protein+CII *p.lyt.cII* simulations, Lyt and CII are produced in synchrony, reflecting their co-transcription. The *p.lyt/p.cII* column shows the results when these productions are separate reactions. For the *cII shut-off* column, Lyt and CII are produced synchronously up to the first replication after which time CII production ceases.

The addition of noise destroyed the ability of almost all of the two-protein networks to perform 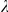-like counting. The left panel of Figure 2 shows the 9 most successful two-protein motifs for the deterministic counting and memory task, and gives the number of parameter sets that worked in each case [16]. These networks comprise all possible variants of the core Lys-Lyt mutual repression motif and include over 95% of successful networks. The three noise-resistant two-protein networks contain Lys positive autoregulation and one of these also has Lyt negative autoregulation, the regulatory motif seen for 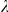 CI-Cro (Figure 2).

**Figure 3:**
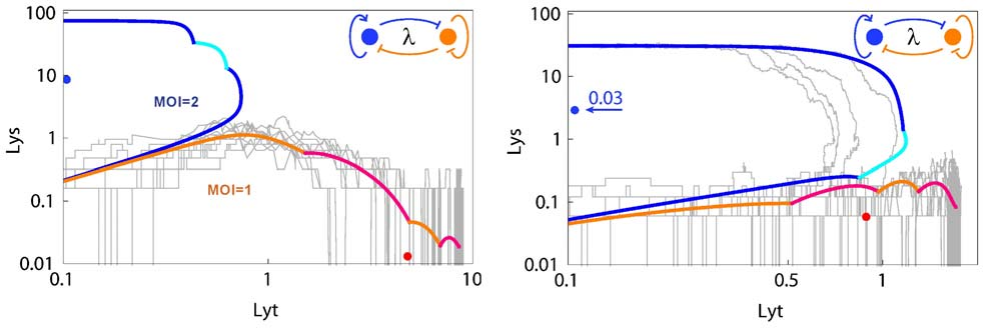
Trajectories of Lys and Lyt concentrations for two-protein networks. The left panel shows a rare two-protein network performing the decision task in the presence of noise. The right panel shows a network that performs the deterministic task but fails in the presence of noise. Lyt and Lys concentrations are given in units of each protein's binding constant for regulation of the opposing protein. Red-orange trajectories depict deterministic simulations of MOI = 1 infections, leading to lysis. Blue-cyan trajectories depict deterministic simulations of MOI = 2 infections, leading to lysogeny. The color of the trace changes at each genome replication time. Grey trajectories are 10 examples of standard stochastic simulations of MOI = 1 infections. Dots show the N = 1 steady states (single copy immune and anti-immune states).

The left panel of Figure 3 shows the behaviour of one of the rare successful 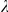-like 2-protein networks. In the deterministic simulations, the trajectory of the Lys and Lyt concentrations in the MOI = 1 case (red-orange trace) diverges rapidly from the trajectory in the MOI = 2 case (blue-cyan trace), so that by the first replication (marked by a colour change) the Lys/Lyt concentrations are very different. The Lys/Lyt trajectories for 10 stochastic simulations for the MOI = 1 case show considerable variation from infection to infection but nevertheless reliably progress towards Lyt dominance over Lys (lytic development). In general, noise caused networks to fail to reliably choose lytic development in MOI = 1 infections (right panel, Figure 3). The two-protein networks use subtle MOI-dependent differences in Lys and Lyt production and stabilities (a ‘CI-Cro battle’) in the period soon after infection and before the first replication [16], and the low protein numbers at this time make the networks particularly susceptible to noise.

Thus, although it is *possible* for a two-protein 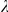-like network to perform the 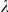 counting task in the presence of noise and in the absence of a CII-like function, it would likely be difficult for evolution to ‘find’ the appropriate parameters for this motif. Furthermore, the few successful two-protein networks fail with small increases in the level of noise.

### CII gives noise resistance

The addition of a CII-like function that activates Lys production and is co-transcribed with Lyt significantly improved the fraction of networks able to function in the presence of noise (Figure 2, right panel *p.lyt.cII*). For the simplest two-protein+CII motif, where there is no direct autoregulation by Lys or Lyt (motif G, Figure 2), 8% of the networks that performed the deterministic task were noise-resistant. Similar fractions of noise resistant networks were seen for all motifs except those in which Lyt positively regulates itself (motifs A, D, and E in Figure 2); it is interesting that this is the only regulatory link that is not present in 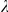.

**Figure 4:**
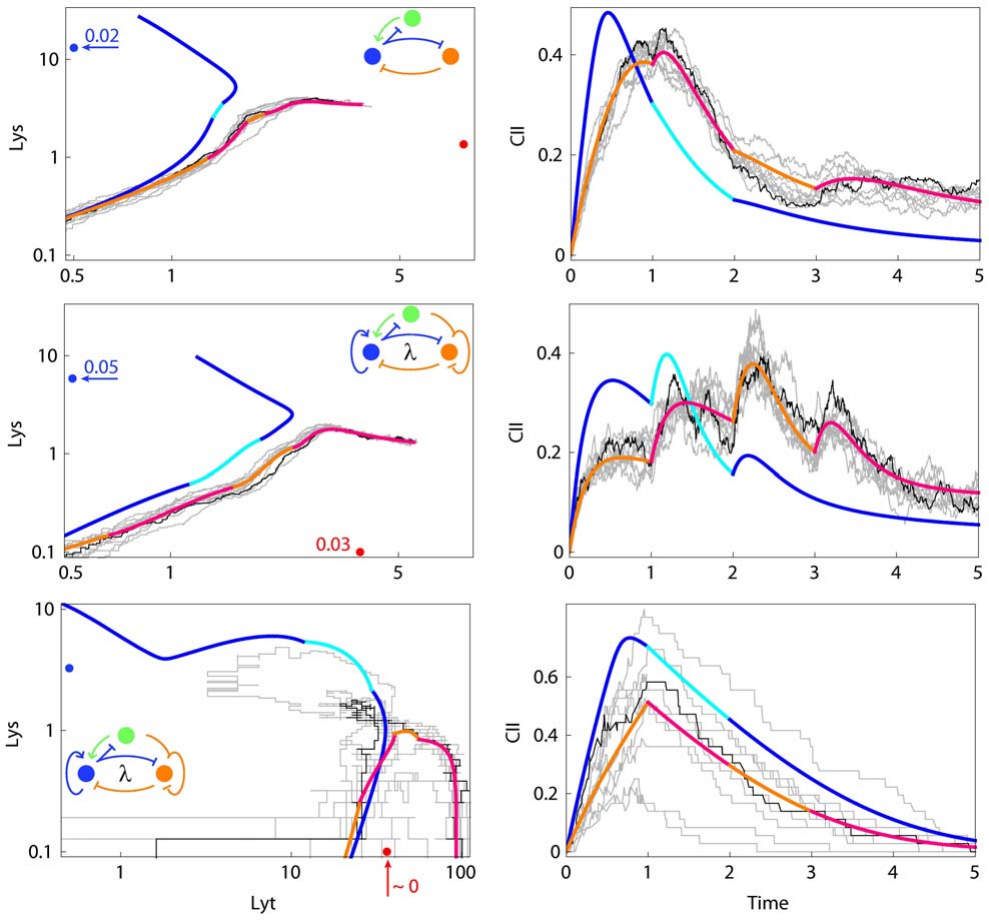
Examples of two-protein+CII networks that can perform the decision task in the presence of noise. In each network 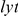 and 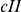 are co-transcribed. The upper two networks (*p.lyt.cII*) work at the standard 6% noise level, while in the lower network CII production is shut off after the first replication (*cII shut-off*). This network functions at a 20% noise level. The left panels are as in Figure 3. The right panels show the time development of CII. All times are shown in units of the phage genome replication time, whereas CII levels are shown in units of its binding constant for activation of Lys protein production.

The upper two pairs of panels in Figure 4 show the Lys/Lyt trajectories and the CII time courses for two of these networks. Initial CII production is proportional to MOI, setting the ground for the robust separation of the Lys-Lyt trajectories in the MOI = 1 and the MOI = 2 cases. Repression by Lys (upper network) or by Lys and Lyt (middle network) limits CII production and results in a reduction in CII levels as infection progresses.

Why does CII provide noise-resistance? We believe that the two-protein networks are highly constrained because both Lys and Lyt must have dual functions as ‘deciders’ and ‘maintainers’. Adding CII relieves Lys of the lysogenic decider role, allowing Lys to be optimized for lysogenic maintenance. A critical property of the lysogenic decider is a short half-life, which means that any of the lysogenic decider that is made before the first replication in the MOI = 1 case quickly decays and does not thwart lytic development once the genome copy number increases [16]. The pressure for a short CII half-life seems to be even stronger in the presence of noise; the mean lifetime of CII decreases from 0.8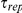 in the networks successful in the deterministic task to about 0.4 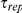 for those passing the stochastic test. In contrast, the lysogenic maintainer works better with a long half-life. The mean lifetime of Lys in the 2-protein networks passing the deterministic task was 1.2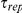, and this increased to 4.6 when CII was added [16]. This increase in half-life means that Lys approaches its steady state more slowly. This means that Lys can have reduced power in the rapid decision phase, in order to avoid interfering with the Lyt-CII battle, while retaining its effectiveness in the maintenance phase. A long half-life reduces noise in Lys levels due to time-averaging over a longer period, and should thus stabilize the commitment to lysogeny, since downward fluctuations in Lys levels in MOI = 2 infections would lead to upward fluctuations in Lyt and could thus cause transitions to lytic development. Lambda CI is stable [30], while CII has a half-life of only ca. 2 min [28].

### Correlation of Lyt and CII production is important for noise resistance

To simulate co-transcrption of the *lyt* and *cII* genes in the *p.lyt.cII* networks we made the production bursts of Lyt and CII proteins occur together (see Methods), reflecting simultaneous translation of both genes from the same unstable mRNA molecule. We expected that this would make the networks more noise resistant. Lyt and CII function as opposing deciders and thus the *ratio* of these proteins is critical. In the MOI = 2 infection CII must dominate Lyt, but in the MOI = 1 infection Lyt must dominate CII. To test the importance of co-transcription, we carried out Lyt and CII protein production in separate reactions in the simulation, as if they were transcribed from distinct, though identically regulated promoters (*p.lyt/p.cII*). Thus on average their production was identical to the *p.lyt.cII* case, however their stochastic bursts of production were uncorrelated.

Removal of correlated Lyt-CII production prevented two-thirds of the networks from functioning (Figure 2). Thus, in the initial Lyt-CII battle that makes the decision, it is indeed important that there are no random production events of CII proteins without a corresponding production event of Lyt proteins, and vice versa.

Co-transcription of a Cro-like function and a CII-like function is widespread in temperate phages [31]–[35], suggesting that reducing fluctuations in the ratio of these proteins may be important in the lysis-lysogeny decision processes of many such phages.

### Noise resistance is aided by rapid shut-off of CII

Despite this benefit of co-transcription of Lyt and CII, it also creates difficulties once the lytic pathway is chosen, because Lyt transcription must be maintained and this results in continued CII production. In the CII trajectory plots of the MOI = 1 *p.lyt.cII* network infections, we noticed that CII levels stabilize at concentrations which are a considerable fraction of its maximal concentration (see the upper two networks in Figure 4). The presence of this CII de-stabilizes the lytic pathway because fluctuations in CII can over-stimulate Lys production and cause stochastic passage to lysogeny, the predominant failing of the networks. The drop in CII levels later in lytic development is due to repression by Lys, sometimes aided by repression by Lyt. However, these repressions cannot be strong, otherwise Lyt levels become too low.

We expected that mechanisms to remove CII without affecting Lyt levels would help the networks resist noise. This may be achieved in part by the short half-life of CII but additional mechanisms could be useful. We therefore tested a modified regulation where CII production was simply terminated after a single phage genome replication time. This change almost doubled the number of networks able to perform the stochastic task (*cII shut-off* Figure 2). A few of these networks (8 of 211) are able to function in the presence of a significantly higher level of noise (see Methods). The lower panel of Figure 4 shows one of these networks at high noise levels. The timing of the *cII* shut-off is not critical; we found similar results if CII production was stopped after two phage genome replication times instead of one.

Accordingly, our simulation predicts that there should be some extra mechanisms to reduce the level or activity of CII in the 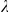 network. Several experimental observations support this prediction. Measurements of CII activity and protein levels show a pulse in the initial stage of infection, with the decrease dependent on the presence of Cro and CI [20]. Direct reduction of *cII* transcription by CI and Cro by repression of the lytic 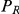 promoter would account for some of this decrease. However, CII activity also depends in indirect ways on the activity of the other lytic promoter, 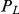, which is also repressed by CI and Cro [36]. The CIII protein, which is expressed from the 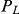 operon, protects CII from degradation and very little lysogeny occurs in its absence [37]. The N anti-terminator protein, is made by the first gene expressed from 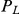. N antitermination of lytic transcription increases expression of both CII and CIII. Like CII, N has a short half-life [38], thus repression of 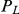 by Cro and CI should quickly reduce N levels, leading to reduced CII and CIII expression and decreased CII stability. CII activity is also controlled by temperature shift and by SOS activation in ways that are not yet well understood [39] and these controls might also be partially active during normal infections. This, it is plausible that a number of compounding effects on CII could cause the sharp decrease in its activity that our analysis predicts would stabilize lytic development.

Another way to avoid residual CII activity interfering with lytic development would be to enter a phase that is resistant to CI, such that even high CI levels cannot block lysis. Unlike lysogeny, 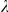 lytic development is a transient state and does not require a stable anti-immune state for its completion [40]. The existence of some irreversible commitment step in lytic development, apparently acting *in cis*, is suggested by recent experiments [21].

### Concluding remarks

Our analysis suggests that a number of features of the 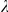 circuitry contribute to a reliable, noise-tolerant decision in response to the multiplicity of infection.

One major strategy is dividing the tasks of establishing lysogeny and maintaining lysogeny between two proteins. This allows CII to be unstable, a feature necessary for the rapid decision, and allows CI to be stable, reducing noise in the commitment to lysogeny. Division of labour between deciders and maintainers seems likely to be a general strategy for decision circuits. As well as allowing separate optimization of the two functions, it means that the decider factors can be removed once the decision is made, in order to avoid them interfering with maintenance. As pointed out by Gann [41], this approach is used by the *Drosophila* sex-determination pathway, where the action of the ‘decider’ proteins (the numerator and denominator functions that count the ratio of the sex chromosomes and autosomes) is developmentally limited [42].

Another important strategy is co-transcription of *cro* and *cII*, which correlates fluctuations in these competing decider functions to reduce noise in their ratio. Co-transcription is common in antitoxin-toxin systems [43] and presumably reduces noise in order to ensure that the toxin is never accidentally in excess of the antitoxin.

Remarkably, our requirement of noise resistance for possible CI-Cro-CII like networks pinpoints abilities of CII that indeed conform to CII properties in lambdoid phages: CII is short lived, CII is produced with Cro from polycistronic mRNA and furthermore CII production is confined to a short time interval after infection. Therefore our analysis suggests that the present understanding of CII regulation and its functional role in the 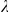 decision is reasonably complete.

## Methods

The stochastic simulations were implemented through the Gillespie algorithm [44], that works by interpreting production and degradation rates as probabilities per unit time for production and degradation events. The production and degradation rates we take to be the same as those occuring in the deterministic equations of [16]: 
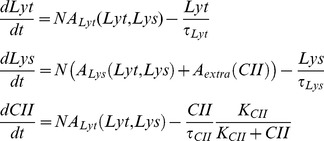
(1)where 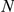 is the current phage genome count (doubling at each phage replication time). In the above equations the production activities 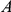 depend on the chosen parameters, that for instance, for a 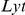 self activation and repression by 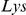 would take the form 
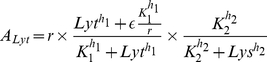
(2)where 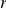 is a rate constant and 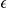 is the ‘leak’, the minimum activity of the given promoter, which we typically keep very small. Thus, each two protein GRN has the following parameters: 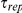, 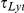, 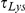, 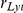, 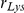, 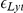, 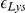 plus two parameters for each interaction link, the Michaelis-Menten constant 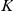 and the Hill coefficient 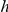. For the two-protein+CII GRNs we have five additional parameters, setting the saturated decay of CII and its regulation of Lys production.

In the stochastic simulations, transcription and translation are not modelled in detail, rather the protein levels are incremented and decremented with a fixed step, 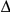, at each change. The time for the next change is randomly chosen from a Poisson process with mean time between events given by the reciprocal of the sum of the production and degradation rates at the given time (positive and negative terms in the equations in 1) divided by their respective 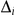
s. We chose to have separate incrementation steps 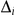 for each protein level, in order for all proteins to have the same standard deviation to mean (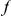) of the total production at maximal rate within one phage genome replication time and one phage genome present.

The standard level of noise in the simulations was set by choosing a 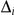 for all involved proteins that secures a 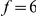
% noise level at their maximum production during one phage replication 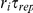. We also tested higher noise levels, and found systematically lower success frequency for all motifs.

The average number of production events of protein 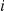 in the time interval 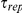 is therefore 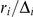, which for a Poisson process is equal to the variance in the number of events. Thus 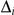 is fixed by the noise through 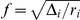. By defining noise in terms of maximal production level, the step-sizes will be the same for different motifs that share the same parameters. Therefore one finds that a particular motif architecture can reduce or enhance the effect of the noise.

For comparison, in phage 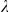 one expects about 300 CI molecules in a lysogen. Given that 1–5 CI proteins are produced per message [45], and that 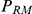 is repressed by a factor 3 in a normal lysogen [46], the CI production noise should be 5%–10%. Direct measurement of 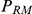 activity fluctuations in RexA- mutants of 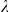 indicates a noise level of 30% [47]. The promoter for Cro and CII is about as strong as a maximally active promoter for CI [27], which suggests similar noise levels for CI. Given that these relatively high noise levels are associated to production over a full bacterial generation, the unregulated noise level after a 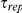 of about 5 min may be even higher. This produces severe constraints on the circuit design due to the unavoidable noise associated with low levels of regulatory proteins.

In the standard two-protein+CII networks, co-transcription of their lyt and cII genes (*p.lyt.cII*) was simulated by producing the Lyt and CII proteins in synchrony, though the size of the bursts could be different for the two proteins. This produces a correlation of Lys and CII production that is probably slightly stronger than seen for genes which share the same mRNA but are translated independently. In addition, in 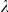 some extra decorrelation occurs because some Cro is produced from a shorter mRNA that terminates before reaching cII. In the separately transcribed simulations (*p.lyt/p.cII*), the Lyt and CII proteins are produced as separate reactions.

Each of the successful networks found in the deterministic screening [16] was subjected to 100 stochastic simulations of single and double infections. To decide whether each infection chose the lytic or lysogenic pathway, we used the end states in Lys-Lyt space of the deterministic MOI = 1 (lytic) or MOI = 2 (lysogenic) simulations as targets. The N = 8 end states were those attained after replication to N = 8 followed by 20 generations without replication. The N = 1 end states were those attained by starting from the N = 8 end states, reducing N to 1 and simulating for 80 generations [16]. For a successful deterministic network the Lys/Lyt ratios in the MOI = 1 and MOI = 2 end states had to be different by a factor of at least 10 [16]. The stochastic simulated infections were scored as going lytic if the N = 8 and N = 1 end states in Lyt-Lys phase space were closer to the deterministic MOI = 1 end states than to either of the deterministic MOI = 2 end states. Similarly, the infection was scored as lysogenic if the end states were closer to the deterministic MOI = 2 end states.
